# SUB-Immunogold-SEM reveals nanoscale distribution of submembranous epitopes

**DOI:** 10.21203/rs.3.rs-3876898/v1

**Published:** 2024-01-22

**Authors:** Katharine K. Miller, Pei Wang, Nicolas Grillet

**Affiliations:** 1 Department of Otolaryngology-Head & Neck Surgery, School of Medicine, 240 Pasteur Drive, Stanford University, Stanford, CA 94305, USA

## Abstract

Electron microscopy paired with immunogold labeling is the most precise tool for protein localization. However, these methods are either cumbersome, resulting in small sample numbers and restricted quantification, or limited to identifying protein epitopes external to the membrane. Here, we introduce SUB-immunogold-SEM, a scanning electron microscopy technique that detects intracellular protein epitopes proximal to the membrane. We identified four critical sample preparation factors that contribute to the method’s sensitivity and validate its efficacy through precise localization and high-powered quantification of cytoskeletal and transmembrane proteins. We evaluated the capabilities of SUB-immunogold-SEM on cells with highly differentiated apical surfaces: (i) auditory hair cells, revealing the presence of nanoscale Myosin rings at the tip of stereocilia; and (ii) respiratory multiciliate cells, mapping the distribution of the SARS-CoV-2 receptor ACE2 along the motile cilia. SUB-immunogold-SEM provides a novel solution for nanoscale protein localization at the exposed surface of any cell.

Understanding the cellular and subcellular distribution of proteins is crucial for unraveling their potential physiological functions and mechanisms of action, particularly in the context of proteins involved in pathologies. Super-resolution microscopies allow *in situ* protein localization below the diffraction limit, reaching 350 nm of axial resolution by pixel reassignment (Airyscan)^1^ (Extended Data Fig. 1a), down to 70 nm for stimulated emission depletion (STED)^2^, and 50 nm for advanced single-molecule localization microscopy (DNA paint)^3^. Because proteins are around 1–10 nm in size, fine mapping of protein location *in situ* requires better resolution^4,5^. Higher resolution of protein localization is achieved by immunogold electron microscopy of embedded and sectioned samples (1–3 nm on 2D) when a primary antibody labels a protein epitope and is then detected by a secondary antibody conjugated to a gold bead^6^. However, conventional techniques like immunogold transmission electron microscopy (TEM) or focused-ion beam SEM (FIB-SEM), are highly time-consuming and often impractical for studying large sample sizes, limiting quantification and, therefore, the significance of results. For surface-exposed cells, immunogold-SEM is an alternative; upon SEM imaging, the gold-conjugated secondary antibodies generate more backscattered electrons (BSE) than the surrounding cell surface and can be imaged with a BSE detector at a 0.5-nm resolution^7^. However, this protein localization approach has been restricted to extracellular epitopes on the exposed cell surface of tissue^7^. Although effective for localizing extracellular epitopes, such as stereocilin^8^, Cadherin23^9,10^, Protocadherin15^9,10^, and PKHD1L1^11^, in auditory hair cells, it fails when applied to submembranous epitopes. Thus, no technique produces nanometric resolution for proteins located below the cellular membrane that allows large-scale sampling and high-powered statistical analysis.

Here, we systematically investigated the standard immunogold-SEM procedure, identified critical steps required for submembranous protein detection, and improved them to reach consistent results across protein and tissue types. We found four critical parameters that, when combined, allow the robust detection of submembranous epitopes. We named this method “SUB-immunogold-SEM” (Fig. 1a). Additionally, we (i) developed an alternative permeabilization step for cytoplasmic proteins farther from the cell surface, (ii) demonstrated its applicability to any cell with an exposed surface, (iii) showcased the potential for double protein-labeling, and (iv) illustrated the strength of large-scale quantification by revealing the nanoscale distribution patterns of proteins involved in diseases along actin- and microtubule-based membrane protrusions.

## RESULTS

### Development of an immunogold-SEM method for submembranous epitope detection

**Fig. 1:**
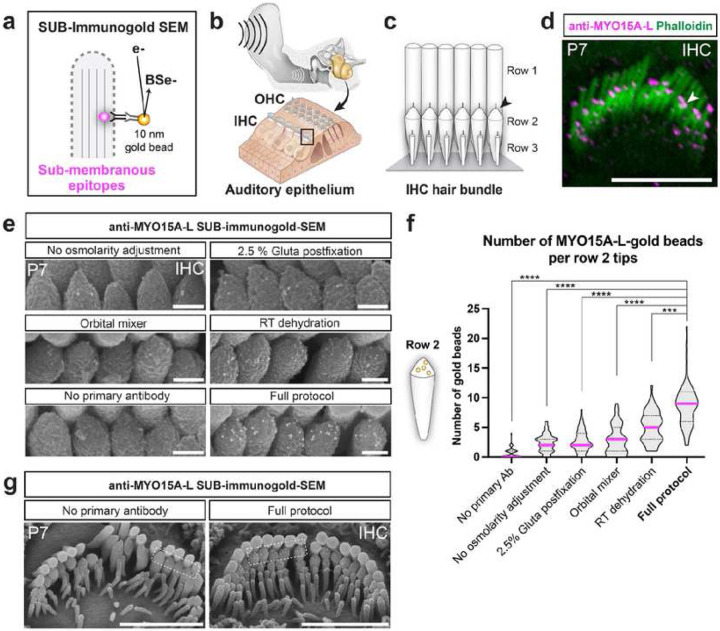
Development of the SUB-immunogold-SEM method to detect submembranous epitopes. **a,** Schematic illustrating the SUB-immunogold-SEM principle, where gold-bead conjugated secondary antibodies are detected at the sample surface as strong sources of backscattered electrons (BSe-). **b,** Graphic depicting the main test tissue, the sensory auditory epithelium located within the cochlea of the inner ear, housing the mechanosensitive sensory cells, the inner (IHCs) and outer hair cells (OHCs). **c**, Cartoon illustrating the apical surface of IHCs, where the hair bundle, the organelle of sound mechanotransduction, is situated. The hair bundle is made of actin-filled membrane protrusions called stereocilia arranged in rows of increasing heights and connected by external filaments. **d**, Whole-mount immunofluorescence staining of a P7 IHC hair bundle with anti-MYO15A-L, with phalloidin staining the stereocilia. The MYO15A-L signal forms puncta at the tip of row 2 stereocilia, where the auditory mechanosensitive ion channels are located (arrowhead). Scale bar = 6 μm. **e**, Each panel is a close-up micrograph of P7 IHC row 2 tips after distinct SUB-immunogold-SEM protocols, each modified in a single step of the method. Secondary antibodies were conjugated to 10-nm gold bead. Scale bar = 200 nm. **f**, Quantification of the number of MYO15A-L gold beads per row 2 tip in each condition, represented in a violin plot with the median in pink and quartile in dashed lines. Per group: n_cells_ = 10, n_stereocilia_ ≥ 55. One-sided Mann-Whitney U tests were performed to compare a given modified protocol with the full protocol. ****, P ≤ 0.0001. See Extended Data Table 1 for full group and statistical description. **g**, SUB-immunogold-SEM on P7 IHC hair bundles comparing the full protocol using the anti-MYO15A-L antibody with no primary antibody control. The dashed boxes in e represent the zoomed area showed in e. Scale bar = 2 μm.

The standard immunogold-SEM procedure involves multiple steps: First, the tissue of interest is lightly fixed with aldehydes and dissected, and permeabilized and blocked to allow antibody penetration and reduction of non-specific binding. Next, immunostaining is performed with a primary antibody recognized by secondary antibodies attached to gold beads (Extended Data Fig. 1b). Following strong post-fixation with glutaraldehyde and paraformaldehyde to stabilize the signal, the sample undergoes processing for SEM imaging. This process includes progressive dehydration to preserve the sample structure under the high-vacuum condition of the SEM chamber and metal coating (palladium, in this case) for electrical conductivity. During SEM imaging, the electron beam landing on the sample generates two main kinds of electrons which can be mapped spatially using dedicated detectors: (i) secondary electrons, emitted from the stimulated atoms at the sample surface, which provide topologic information; and (ii) backscattered electrons (BSE), generated when incident electrons that encounter nuclei of the sample atoms and are reflected by them. The denser the atom nuclei, the more BSE will be generated. Therefore, BSE imaging provides atomic (Z) information on the sample surface composition, enabling a strong signal contrast between atoms of gold (Z = 79) vs. palladium (Z = 46). We tested whether the standard immunogold-SEM procedure used previously for external epitopes would allow for the detection of internal ones^12^. As a test tissue, we used the sensory auditory epithelium found within the cochlea of the inner ear, which contains the sound-detecting sensory cells, the hair cells (Fig. 1b). Inner hair cells (IHCs) provide auditory information to the brain, whereas outer hair cells (OHCs) permit cochlear amplification and sound selectivity (Fig. 1b). The apical surface of hair cells forms hair bundles, assemblies of actin-filled membrane protrusions known as stereocilia arranged in rows of increasing heights, which are the mechanosensitive organelles critical for hearing (Fig. 1c). Owing to the small dimensions of the stereocilia (200–450 nm in width, 1–6 μm in height^13^) and their highly compartmentalized protein localization^14^, this is an ideal tissue to evaluate the capabilities of our method. To test the protein with submembranous localization, we chose MYO15A-L (Long isoform), a cytoskeletal protein with submembranous localization at the tip of stereocilia equipped with the auditory mechanosensitive ion channels and for which a specific antibody has been generated (antibody PB888^15^).

Furthermore, in previous post-embedding immunogold-TEM studies, MYO15A-L was detected in close proximity to the surface of row 2 stereocilia with a distance estimated at 10–16 nm from the micrographs obtained at P16 IHC^16^. To establish the specificity and expression pattern of this antibody, we conducted high-resolution Airyscan immunofluorescence on mouse IHCs at P7. Phalloidin was employed to label the stereocilia cytoskeleton (Fig. 1d)^17^. As expected, little signal was detected in the tallest stereocilia (row 1), whereas the transducing stereocilia tips (row 2 stereocilia and likely the smaller rows, which are poorly stained by phalloidin) present a strong punctiform signal (Fig. 1d). We then replaced the fluorescent secondary antibody with 10-nm gold-bead conjugated secondary antibodies and processed samples for immunogold-SEM. We identified four critical factors in the immunogold-SEM procedure that were indispensable to maximize the detection of submembranous proteins by SEM and optimized them.

The first critical factor concerned tissue preservation. We found that the osmolarity of the solution used for the initial tissue extraction and the dilution of the concentrated fixative (1:8) needed to be adjusted to 310 mOsm. When the osmolarity of the solution was not adjusted (287 ± 2 mOsm for Hank’s Balanced Salt Solution [HBSS]), the number of MYO15A-L gold beads per stereocilia tip was low with 2.4 ± 1.5 on average (Fig. 1e, f). Second, to increase the resistance of the immunogold staining on the sample, we performed the post-fixation step with 10% Glutaraldehyde for 24 h at 4 °C. Post-fixation as in the standard procedure with 2.5% Glutaraldehyde showed that the number of MYO15A-L gold beads per row 2 stereocilia tip was low, with 2.7 ± 1.8 on average (Fig. 1e–f). The third critical factor highlighted the importance of gentle nutation during immunostaining steps for optimal mixing of reagents with the sample, improving submembranous epitope detection most likely because nutation allows optimal mixing of the reagents with the sample and prevents sedimentation of gold-conjugated antibodies. Use of orbital shaking showed that the number of MYO15A-L gold beads per row 2 stereocilia tip was low, with 2.8 ± 2.4 on average (Fig. 1e,f). The fourth critical factor helped maintain the immunogold signal on the sample during the harsh treatments required for SEM sample preparation: To be suitable for imaging by SEM, samples must withstand the high-vacuum conditions within the SEM chamber, necessitating a dehydration process^18^. Sample dehydration is achieved by progressively replacing water in the sample with alcohol (ethanol), which is then replaced by liquid CO_2_ (LCO_2_). In a critical point dryer chamber, the LCO_2_ is transitioned to its supercritical fluid state (31 °C; 1070 psi) where it fluctuates between gas and liquid states and is gently vented out of the sample without affecting the sample’s ultrastructure. However, the alcoholic dehydration also removes lipids from the sample, which could potentially contribute to the detachment of the immunogold staining from the sample. To limit the extent of lipid extraction during dehydration, we performed 15% to 100% ethanol incubations at ice-cold temperatures. Incubations at room temperature (RT) showed that the number of MYO15A-L gold beads per row 2 stereocilia tip increased compared with conditions without the other critical factor, albeit remaining relatively low at 5.1 ± 2.6 on average (Fig. 1e,f). However, upon optimization and combining all these factors, the number of MYO15A-L gold beads per row 2 tip reached an average of 8.8 ± 3.7 (Fig. 1e,f). Notably, consistent staining was achieved in all row 2 stereocilia of all IHCs (Fig. 1g).

In conclusion, consistent detection of submembranous epitopes through immunogold-SEM requires the integration of four parameters derived from the conventional protocol for detecting external epitopes (Extended Data Fig. 1c). These modifications enhance tissue preservation, the immunogold-staining reaction, and the maintenance of the immunogold reaction at the sample surface. We coined the term “SUB-immunogold-SEM” for this optimized method.

### Detection of intracellular epitopes of transmembrane protein

Considering that the SEM sampling procedure extracts a portion of the plasma membrane, we pondered the suitability of the SUB-immunogold-SEM protocol for detecting intracellular epitopes of transmembrane proteins. Therefore, we attempted to detect the multi-transmembrane protein ATPase plasma membrane Ca^2+^ transporting 2 (ATP2B2 or PMCA2). The mouse *Atp2b2* gene produces different splicing isoforms, including isoform PMCA2a, which is abundant at the stereociliary membrane of OHCs and IHCs, albeit at a much lower level^19,20^.

**Fig. 2:**
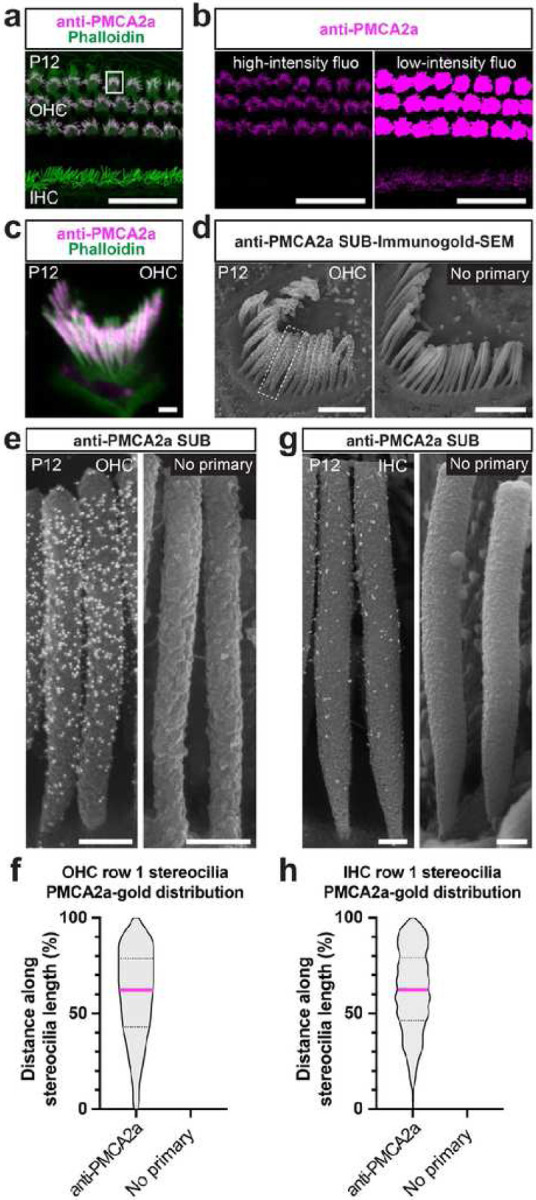
Detection of intracellular epitopes of the transmembrane protein PMCA2a by SUB-immunogold-SEM. **a**, Whole-mount immunofluorescence staining of P12 mouse auditory epithelium displaying the single row of IHCs and the three rows of OHCs hair bundles labeled with phalloidin, while PMCA2a preferentially stained OHC hair bundles. Scale bar = 2 μm. **b**, PMCA2a is also present at much lower levels in IHCs. The fluorescence intensity needed to visualize this labeling saturates the OHC fluorescence signal. Scale bar = 2 μm. **c**, The posterior aspect of a P12 OHC shows PMCA2a staining along the row 1 height. Scale bar = 1 μm. **d**, SUB-immunogold-SEM on P12 OHC hair bundles comparing PMCA2a antibody labeling with no primary antibody control, with 10-nm gold-bead conjugated secondary antibody. Scale bars = 1 μm. **e**, High-magnification view of P12 OHC row 1 stereocilia imaged after anti-PMCA2a SUB-immunogold-SEM, or SUB-immunogold-SEM without the primary antibody. Scale bars = 200 nm. **f**, Distribution of the PMCA2a-gold position relative to the stereocilia length (expressed in %). Per group: n_cells_ ≥ 6; n_stereocilia_ PMCA2a = 30, no primary = 23. **g**, High-magnification of P12 IHC row 1 stereocilia imaged after anti-PMCA2a SUB-immunogold-SEM or SUB-immunogold-SEM without the primary antibody. Scale bars = 200 nm. **h**, Distribution of the PMCA2a-gold position relative to the stereocilia length (expressed in %). Per group: n_cells_ ≥ 7; n_stereocilia_ PMCA2a = 28, no primary = 9. See Extended Data Table 1 for a full group description.

Using a validated antibody recognizing a PMCA2a intracellular epitope (F2A^19^), we confirmed these results through immunofluorescence on apical P12 WT cochlea. While the signal in OHC hair bundle was intense, detection of IHC signal required a longer exposure, saturating the OHC signal (Fig. 2a–c). SUB-immunogold-SEM for PMCA2a on littermates and imaging the IHC hair bundle from the back revealed an average of 182 ± 49 PMCA2a-gold beads covering each OHC row 1 stereocilia backside, while the negative control (omitting the primary antibody) yielded no signal (Fig. 2d). Interestingly, we observed a heterogenous distribution of PMCA2a-gold beads along the height of row 1 stereocilia (Fig. 2e). Measuring the position of the PMCA2a-gold beads from the base with nanometric precision and normalizing to stereocilia height (six apical OHCs, 30 row 1 stereocilia, 5644 PMCA2a-gold beads) (Fig. 2f) revealed low density at the stereocilia base (10% of the height), and PMCA2a-gold beads were present at a low density. Moving upward to about 70% of the stereocilia height, bead density increased, plateauing until the last 10% of stereocilia height, where it dropped again. In conclusion, our suspicion of a heterogeneous distribution of PMCA2a along the OHC stereocilia membrane was confirmed by our quantification. Additionally, these results aligned with an independent study where anti-PMCA2 immunogold-TEM on P26 rat OHC showed a stronger PMCA2 signal at row 1 stereocilia shaft compared with the stereocilia base and tip^20^.

We then investigated whether the heterogeneous PMCA2a pattern was also present in IHCs. Owing to PMCA2a’s low density along the IHC stereocilia height, a heterogenous pattern was not initially evident from observation alone and therefore required quantification (Fig. 2g). We detected an average of 79 ± 23 PMCA2a-gold beads along P12 row 1 IHC (Fig. 2h). The distribution of PMCA2a-gold beads along IHC row 1 stereocilia (eight apical IHCs, 28 row 1 stereocilia, 2294 PMCA2a-gold beads) exhibited low density toward the stereocilia base, progressively increasing to about 60% of the stereocilia height, then stabilizing until the last 10% of the height, where the density decreased, similar to OHCs (Fig. 2h). Overall, the SUB-immunogold-SEM method facilitated the nanoscale detection of a transmembrane protein in hair cell stereocilia. The ease of extensive sampling captured variation in gold-bead density along the stereocilia length, even in limited amounts.

### Enhanced post-fixation and tissue permeabilization for enhanced detection of intracellular stereociliary protein

**Fig. 3:**
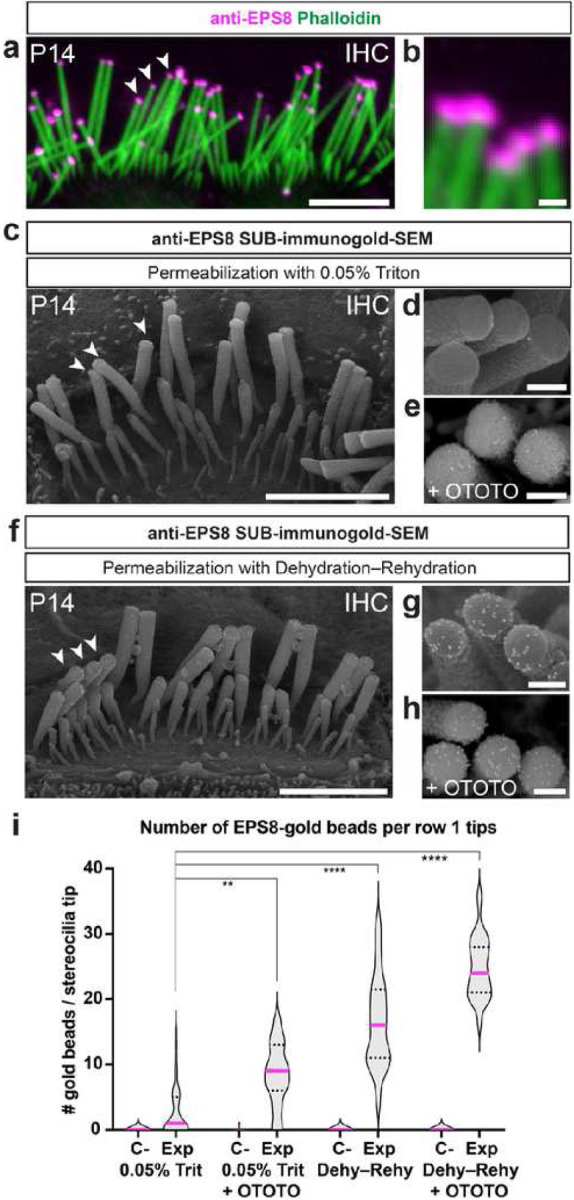
Detection of the membrane-distant protein EPS8 using SUB-immunogold-SEM. **a**, Whole-mount immunofluorescence staining of the P14 mouse IHC hair bundle revealed the presence of EPS8 protein at the tips of row 1 stereocilia (arrowheads). Scale bar = 5 μm. **b**, Close-up of row 1 tip staining. Scale bar = 500 nm. **c**, SUB-immunogold-SEM on a P14 IHC hair bundle employing the full SUB-immunogold-SEM protocol with 0.05% Triton permeabilization with the anti-EPS8 antibody shows minimal labeling at row 1 tips (arrowheads), with 10-nm gold-beads. Scale bar = 2 μm. **d–e**, Close-up of row 1 tip staining, without or with OTOTO post-fixation Scale bar = 200 nm. **f**, SUB-immunogold-SEM on P14 IHC hair bundle with a dehydration–rehydration step for permeabilization using the anti-EPS8 antibody which showed strong staining at row 1 tips (arrowheads) using 10-nm gold beads. Scale bar = 2 μm. **g–h**, Close-up of row 1 tip staining with dehydration–rehydration step, without or with OTOTO post-fixation. Scale bars = 200 nm. **i**, Quantification of the number of gold-beads at row 1 tips per condition. C-: negative control, no primary antibody; Exp: Experiment. Per group: n_cells_ ≥ 7; n_stereocilia_ ≥ 18. One-sided Mann-Whitney U tests were performed to compare a given modified protocol with the full protocol. ****, P ≤ 0.0001. See Extended Data Table 1 for a comprehensive group description.

Subsequently, we sought to ascertain the capability of our SUB-immunogold-SEM protocol to detect proteins situated deeper within the cell, extending beyond the immediate submembranous area. Our focus was on the actin-binding protein EPS8 (Fig. 3), selectively enriched at the tip of IHC row 1 stereocilia at P14 (Fig. 3a, b)^21^. Previous post-embedding immunogold-TEM work detected EPS8 at 42 and 54 nm from the surface of P35 IHC row 1 stereocilia at P14^16^, implying a potentially farther distance from the membrane than Myo15A-L is. Employing our standard SUB-immunogold-SEM protocol on P14 IHCs resulted in merely 2.8 ± 3.8 EPS8-gold beads at the tip of row 1 stereocilia, and almost none in the absence of the primary antibody (Fig. 3c–d, i).

Considering that our standard protocol’s use of the same light permeabilization conditions as immunofluorescence staining (0.05% Triton for 20 min), we hypothesized that the immunogold reaction might be constrained by the limited accessibility of the gold-conjugated secondary antibody to the antigen and its maintenance during the sample dehydration steps. Therefore, we strengthened the sample post-fixation by employing osmium tetroxide (OsO_4_). OsO_4_ is a strong oxidizer that creates covalent bonds with proteins and lipids. One major limitation of using osmium for SUB-immunogold-SEM application is that it affects the BSE atomic contrast, as osmium (Z = 76) and gold (Z = 79) atoms have similar nuclear composition. Nevertheless, when the sample was postfixed with sequential baths of OsO_4_ and Thiocarbohydrazide (OTOTO)^22,23^, 8.4 ± 4.7 EPS8-gold beads were found at the tip of row 1 stereocilia (Fig. 3e,i). These results suggest that increasing the stability of the immunogold-SEM signal by OTOTO post-fixation maintained the signal (Extended Data Fig. 1d).

Recognizing that EPS8 is not embedded in the membrane and that the final dehydration step could affect immunogold signal maintenance, we explored whether performing a dehydration step before immunostaining could yield improved results. After initial tissue fixation, ethanolic dehydration and rehydration at ice-cold temperatures preceded the standard protocol. Under these conditions, 17 ± 7.0 EPS8-gold beads were found at the tip of row 1 stereocilia (Fig. 3g, i; Extended Data Fig. 2e). Adding an OTOTO post-fixation to the dehydration–rehydration protocol showed that the results were further improved with 25 ± 4.7 EPS8-gold beads found at the tip of row 1 stereocilia (Fig. 3h,i; Extended Data Fig. 1f). In summary, extending the range of detectable internal epitopes in SUB-immunogold-SEM is achieved by incorporating an initial dehydration–rehydration step before immunostaining. Optional OTOTO post-fixation allows for even higher signal preservation throughout the sample preparation process, albeit at the expense of BSE contrast, making gold beads more challenging to identify at the cell surface.

### Other applications for SUB-immunogold-SEM: double staining and detection of CRE-recombined cells by SEM imaging.

**Fig. 4:**
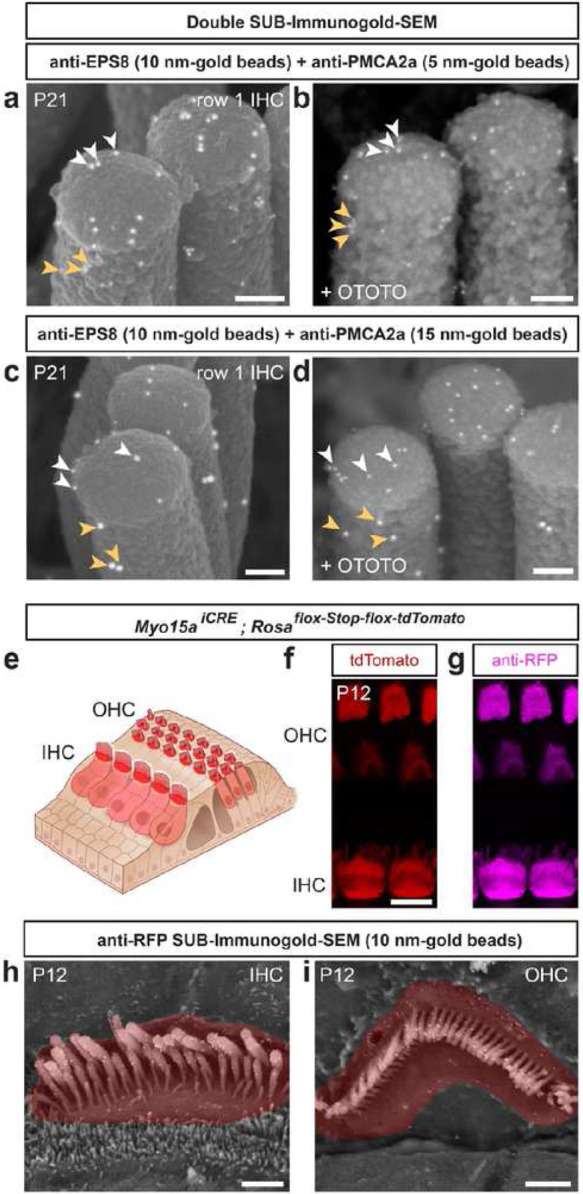
Other applications for SUB-immunogold-SEM: double staining and detection of CRE-recombined cells under SEM imaging. **a–d**, High-magnification of P21 IHC row 1 tips double-stained by SUB-immunogold-SEM for EPS8 (white arrowheads) with 10-nm gold beads and PMCA2 (orange arrowheads) with either 5-nm (a–b) or 15-nm (c–d) gold-beads, without (a and c) or with OTOTO post-fixation (b and d). Scale bars = 100 nm. **e**, Cartoon of the auditory epithelium of a mouse expressing the cytoplasmic red fluorescent reporter tdTomato specifically in hair cells after CRE recombination, used in panels f–i. **f–g**, Direct tdTomato fluorescent signal is detected in OHC and IHC and indirectly via anti-RFP immunofluorescence staining. Scale bar = 8 μm. **h–i**, SUB-immunogold-SEM on P12 IHC hair bundle with a dehydration–rehydration step using the anti-RFP antibody paired with 15-nm gold-bead-conjugated secondary antibody. Labeling is visible at the apical surface of IHC (h) and OHC (i) including the stereocilia (colored in postproduction in red). Scale bar = 1 μm.

By enabling the simultaneous investigation of multiple molecules within a sample, the range of applications for SUB-immunogold-SEM would be significantly expanded. Because both PMCA2 and EPS8 were present in P12 IHC stereocilia, we attempted double SUB-immunogold-SEM experiments first using secondary antibodies conjugated to 10-nm gold beads to detect the anti-EPS8 mouse antibodies and 5-nm gold beads to detect the rabbit PMCA2a antibodies. As expected, larger EPS8-gold beads located at the tip of the row 1 stereocilia, while smaller PMCA2a-gold beads were distributed along the shaft (Fig. 4a). Similar results were found after OTOTO post-fixation (Fig. 4b). We obtained comparable results with larger 15-nm PMCA2a-gold beads (Fig. 4c–d). In summary, double SUB-immunogold-SEM is effective with secondary antibodies conjugated to gold beads of different sizes.

A distinct application for SUB-immunogold-SEM, emphasizing its ability to detect cytoplasmic proteins rather than spatial precision, is the detection of CRE-recombined cells within tissue by SEM imaging. Conventionally, identifying CRE-recombined cells through SEM necessitates correlative light and electron microscopy (CLEM), involving placing cells/tissue on a mesh grid with identifiable landmarks, imaged first using fluorescence then processed and imaged using SEM, ultimately superimposing both images in post-production^24,25^. Thus, CLEM represents a labor-intensive procedure. We attempted to detect the cytoplasmic fluorescent protein tdTomato expressed from the *Rosa26* locus^26^ after hair cell-specific recombination, using the driver allele *Myo15a*^*iCRE*^ (Fig.4e)^27^. At P12, direct tdTomato fluorescence was detected at the apical surface of hair cells, including the stereocilia (Fig. 4f), as was an anti-RFP immunofluorescence (Fig. 4g). SUB-immunogold-SEM with anti-RFP on P12 littermates after dehydration–rehydration permeabilization revealed 15-nm gold beads labeling anti-RFP at the hair cell surface and robustly in stereocilia of both IHC (Fig. 4h) and OHC (Fig. 4i). Consequently, SUB-immunogold-SEM offers a novel alternative method for detecting CRE-recombined cells within a tissue by SEM, enabling simultaneous investigation of their nanoscale morphological characteristics.

### SUB-immunogold-SEM identifies the formation of a nanoscale ring of MYO15A-L molecules at the tips of transducing stereocilia

**Fig. 5:**
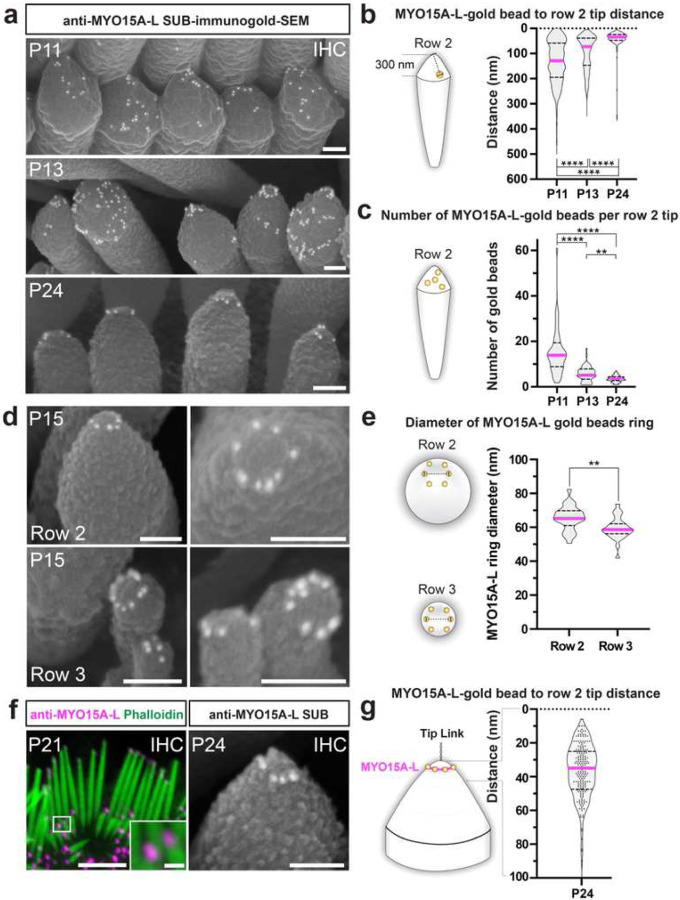
SUB-immunogold-SEM identifies the formation of a nanoscale ring of MYO15A-L molecules at the tips of transducing stereocilia. **a**, SUB-immunogold-SEM on P11, P13, and P24 apical IHC hair bundles using the anti-MYO15A-L antibody with 10-nm gold beads. Scale bars = 100 nm. **b–c**, Quantification of the distance between MYO15A-L gold beads to IHC row 2 tips (b) and the number of gold beads per row 2 tip (c) at different ages. The data are represented in a violin plot with the median in pink and quartiles in dashed lines. Per group: n_cells_ ≥ 4, n_stereocilia_ ≥ 48, n_gold beads_ ≥ 193. To compare multiple ages, nonparametric Kruskal–Wallis tests followed by Dunn’s multiple comparison tests were performed. **, P ≤ 0.01; ****, P ≤ 0.0001. See Extended Data Table 1 for full group and statistical description. **d**, SUB-immunogold-SEM anti-MYO15A-L with 10 nm gold-beads on P15 apical IHC row 2 and row 3 stereocilia tips forming nanoscale rings. Scale bars = 100 nm. **e**, Quantification of row 2 and row 3 MYO15A-L nanoscale rings at P15. n_cells_ ≥ 10, n_stereocilia_ ≥ 25. Two-sided Mann–Whitney U test; **, P = 0.0034. **f**, The MYO15A-L ring at row 2 tips is not discernible by immunofluorescence (scale bar = 3 μm and 300 nm for inset) but is evident from SUB-immunogold-SEM (scale bar = 100 nm). **g**, Focused quantification of MYO15A-L gold-bead distribution (showed in b) for the first 100 nm of IHC row 2 tips. The MYO15A-L row2 ring is positioned 41 ± 39 nm from the stereocilia tip, where the tip link transmitting hair bundle deflection force inserts. n_cells_ = 8, n_stereocilia_ = 56, n_gold beads_ = 193.

After extensively validating the SUB-immunogold-SEM method, we focused on the hair cell expression pattern of MYO15A-L at the nanoscale level during postnatal maturation of the hair bundle. This MYO15A isoform, characterized by a long N-terminal domain, is detected at the tip of IHC transducing stereocilia by immunofluorescence from P4.5 until at least P21.5^14^. Despite the genetic deletion of the MYO15A-L isoform in mice leading to hearing loss and affecting hair bundle morphology and sensitivity to displacement^15,28^, its molecular function remains elusive. To gain more information about its stereociliary localization, we conducted anti-MYO15A-L SUB-immunogold-SEM on apical IHCs at P11, P15, and P24 (n_cell_ ≥ 4; n_stereocilia_ ≥ 48 per group; n_gold beads_ ≥ 193), imaging inside views of the hair bundle (Fig. 5a). We then quantified the distance separating the gold beads from the row 2 tips (Fig. 5b) and the number of gold beads per row 2 tip (Fig. 5c).

**Fig. 6:**
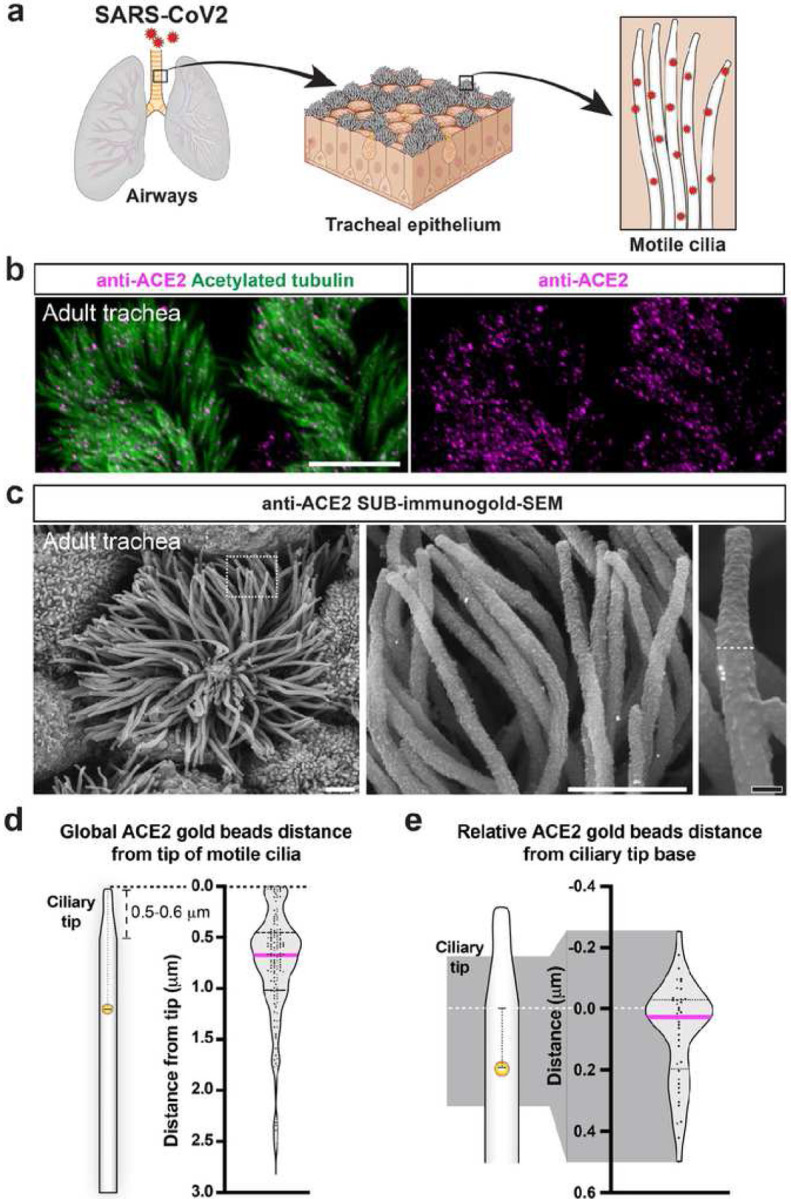
SUB-immunogold-SEM identifies the preferential position of ACE2 SARS-CoV-2 receptor along respiratory motile cilia. **a**, Cartoon illustrating the mouse airways and the tracheal epithelium and its multiciliate cells. The SARS-CoV-2 particles enter the body through the airway and respiratory cells by binding to their motile cilia. **b**, Whole-mount immunofluorescence staining of the mouse adult tracheal epithelium using an acetylated-tubulin antibody labeling the microtubule-rich cilia, along with an anti-ACE2 SARS-CoV-2 receptor antibody. Scale bar = 8 μm. **c**, Anti-ACE2 SUB-immunogold-SEM on a mouse tracheal multiciliate cell with 15-nm gold beads, with a close-up on the ciliary tips. The ciliary tip forms an apical narrow rod that flares into the larger width of the cilia shaft. The base of the ciliary tip is indicated with a dotted line. White scale bar = 1 μm, black scale bar = 100 nm. **d**, Quantification of the distance of all (global) MYO15A-L gold beads along adult tracheal cilia length. Only cilia visible from their tip above 3 μm were included. The ciliary tip has a variable length (0.5–0.6 μm) n_cells_ = 11, n_cilia_ = 125, n_gold_ = 151. **e**, Quantification of the relative distance separating MYO15A-L gold beads from the base of the ciliary tips. The selected pool of gold beads quantified was present from 250 nm above and 500 nm below the ciliary tip. n_cells_ = 11, n_cilia_ = 44, n_gold_ = 44.

We found that the distribution of the MYO15A-L-gold beads within the row 2 tips underwent developmental changes: At P11, MYO15A-L-gold beads were broadly distributed at row 2 tip, within the first 300 nm from the tip (Fig. 5a–b). At P15, a large proportion of MYO15A-L-gold beads (63%) were concentrated within the first 100 nm from the row 2 tip, and at P24, almost all (98%) were contained in this narrow zone (Fig. 5a–b). In parallel, the average number of gold beads visible at row 2 tips from these front views decreased from 16 ± 10 gold beads at P11 to 5.8 ± 3.3 at P13 and 3.4 ± 1.4 at P24 (Fig. 5c). Notably, the arrangement of MYO15A-L-gold beads within the 100-nm zone formed a ring, clearly observable at P15 (Fig. 6d). Measured from top–down views, the diameter of the MYO15A-L-gold beads ring was 65 ± 7.3 nm for row 2 tips and 59 ± 6.6 nm for row 3 tips. In row 2 tips, the distribution of MYO15A-L-gold beads from row 2 tips formed a bell shape, with an average of 41± 39 nm, beyond the resolution of Airyscan super-resolution fluorescence microscopy (Fig. 5f–g).

Thus, MYO15A-L molecules accumulate initially at the tip of transducing stereocilia, forming a ring structure during late developmental maturation between P11 and P24. The ring structure sits below the extreme stereocilia tip, where the tip link inserts and transfers the force induced by sound. In conclusion, from around P15, IHC stereociliary tips exhibit a specialized geometric cytoskeletal organization at the site of auditory mechanotransduction, a revelation unveiled for the first time using SUB-immunogold-SEM.

### SUB-immunogold-SEM identifies the preferential position of ACE2 SARS-CoV-2-receptor along respiratory motile cilia

To demonstrate the applicability of SUB-immunogold-SEM performance in any cell surface exposed cells beyond the cochlea, we focused on the airways with the trachea. The tracheal epithelium contains multiciliate cells (Fig. 6a). The long and thin motile cilia of the multiciliate cells are made of microtubules and contain the angiotensin receptor 2 (ACE2), a cell-surface receptor that binds to severe acute respiratory syndrome coronavirus 2 (SARS-CoV-2) viral particles^29,30^. SARS-Cov-2 binds to the ACE2 receptor and enters the cell, leading to SARS in humans^30–33^. However, how ACE2 receptors distribute along the cilia remains unknown. We used an antibody specific for the short ACE2 cytoplasmic domain^30^ in whole-mount staining of 3-month-old mouse tracheae. While Airyscan super-resolution immunofluorescence revealed acetylated tubulin-positive bundles of numerous thin cilia covering each multiciliate cell (Fig. 6b), the relative position of the ACE2 IF signal was punctiform and colocalized with the cilia. Nevertheless, their relative position could not be determined due to a lack of spatial resolution.

Applying SUB-immunogold-SEM with the same anti-ACE2 primary antibody and 15-nm gold-bead-conjugated secondary antibody uncovered ACE2 gold beads along many cilia of each multiciliate cell, absent when the primary antibody was omitted (Fig. 6c; Extended Data Fig. 2). Because the cilia were generally tangled and densely packed, we rarely viewed their entire length corresponding to 3.45 ± 0.55 μm on average (n_cell_ = 6; n_cilia_ = 18) after SEM sample preparation. Therefore, we mapped the distance of the ACE2-gold beads from the top of a cilium down to 3 μm of its height (n_cell_ = 11; n_cilia_ = 125; n_gold beads_ = 151). While ACE2-gold beads were found all along the 3 μm cilia length, their distribution was not homogenous; a major ACE2 enrichment was found between 400–800 nm from the top of cilia (43% of all gold beads), and to a lesser extent, within the first apical most 50 nm (7% of all gold beads) (Fig. 6d). Interestingly, the apical structural specialization of motile cilia, named the ciliary tip, could be frequently observed by SEM. The ciliary tip externally presented as a progressive thinning of the cilial tip and corresponded internally to the axonemal ending of the 9 peripheral doublet tubules, maintaining the two central tubules^34–36^. The average length of the ciliary tip in tracheal ciliate cells was highly variable, measured at 579 ± 168 nm (n_cilia_ = 54). We speculated on whether the ACE2-enriched position along motile cilia might align with the junction between the shaft and the ciliary tip. Thus, we selected cilia displaying an apparent ciliary tip structure. Our analysis focused on the distribution of ACE2-gold near the ciliary tip junction (250 nm above and 500 nm below the junction) (n_cell_ = 11; n_cilia_ = 44; n_gold beads_= 44) (Fig. 5d). Most of the ACE2-gold beads were located between −100 to +100 nm from the junction (59%) or below it (34%). In conclusion, the SARS-CoV-2 receptor ACE2 does not exhibit a uniform distribution along respiratory motile tracheal cilia. Instead, ACE2 concentrates at the base of the ciliary tip and, to a lesser degree, at the extreme end of the ciliary tip.

## DISCUSSION

SUB-immunogold-SEM introduces a groundbreaking approach for achieving large-scale, quantifiable, and nanoscale localization of submembranous epitopes applicable to any exposed cell surface in living organisms. The direct superposition of the immunogold signal onto the cell surface enables the discovery of unforeseen patterns and structural correlations of scientific significance. Additionally, the quantification of gold-bead position or number can be restricted to a defined area, volume, or structure directly observable at the nanoscale. For example, our quantification reveals the discrete distribution of the SARS-CoV-2 receptor ACE2 relative to ciliary tips, a detail unattainable through light microscopy and time-intensive with immunogold-TEM particularly for equivalent sampling size. The observed low concentration of ACE2 receptor at the ciliary tip aligns with the low number of SARS-CoV-2 particles observed in this zone when SEM was performed on human ciliated nasal cells^32^. The fact that ACE2 is not homogenously distributed along the motile cilia membrane and is instead enriched at the base of the ciliary tip suggests that ACE2 receptor is actively transported by intraflagellar transport, unloaded and accumulated at the end of the 9 doublet tubules. This local ACE2 accumulation could correspond to a selective docking.

Using SUB-immunogold-SEM, we unveiled a distinctive arrangement of a MYO15A isoform within the sound-mechanosensitive bundles of hair cells. This arrangement forms a ring at the tips of stereocilia that host the auditory ion channels. This ring could serve as a cytoskeletal anchor involved in homogenously tensing the pointed stereociliary tip that sits above it. The localization of MYO15A-L had been investigated previously by post-embedding immunogold-TEM^15^. However, the ring pattern could not be seen as its 65-nm width was contained within the ultramicrotome-section thickness^15^. Moreover, the fact that the ring formation occurs only after P11 in mice was unexpected and demonstrated that stereocilia tips are still maturing their cytoskeleton at least until P15. Although row 2 stereocilia were 2.4 times larger than row 3 stereocilia, the diameter of their respective MYO15A-L rings differs only by a factor of 1.1. This result suggests that the structural similarity of transducing stereocilia tip specialization remains largely independent of row identity.

In addition to its unmatched spatial resolution, another notable strength of SUB-immunogold-SEM lies in its capacity for rapid large-scale quantification. However, the dimensions determined during SUB-immunogold-SEM preparation undergo shrinkage because of the sample preparation process. To overcome this challenge and acquire dimensions reflecting living conditions, a conversion is required using factors determined in our previous work^13^. Therefore, the living dimensions of the MYO15A-L ring are as follows: width 94.2 ± 13 nm for row 2 and 85.5 ± 12 nm for row 3, and for row 2, at 62.1 ± 61 nm from row 2 tip.

Furthermore, in a recent report (Wang et al., *submitted*), we capitalize on the sensitivity of SUB-immunogold-SEM to detect the lowly expressed auditory mechanotransduction channel subunits TMC1 at the tips of stereocilia and observed their mislocalization in the absence of another deafness protein, LOXHD1. Finally, an important advantage of this method compared with fluorescence-based ones is that SEM samples can be re-imaged for years.

In conclusion, SUB-immunogold-SEM encourages the re-evaluation of existing data, offering a novel solution for high-resolution protein mapping along the exposed surface of any cell.

## ONLINE METHODS

### Animal models

The Administrative Panel on Laboratory Animal Care (APLAC) at Stanford University (APLAC protocols #28278 and #30305) approved all animal procedures. Mice of both sexes were used in all experiments and were housed in standard Innovive cages with bedding (San Diego, CA, USA). The housing conditions included 12-h light–dark cycles, continuous access to food and water, and a RT maintained at approximately 22 °C. Weekly inspections were conducted to monitor signs of discomfort, and sentinel mice on each rack underwent routine infection testing. C57BL6/J WT mice were purchased from Charles River Laboratories, and the *Myo15a*^*iCRE*^ mouse strain was previously described by Caberlotto et al.^27^. The B6.Cg-*Gt(ROSA)26Sor*^*tm14(CAG-tdTomato)Hze*^/J (Strain #:007914) was obtained from JAX.

### Tissue dissection and fixation

The dissection buffer used was HBSS (Gibco Cat#14175–095) supplemented with 2-mM CaCl_2_ and 0.5-mM MgCl_2_, with osmolarity adjusted to 310 mOsm with D-glucose using an osmometer (Advanced Instruments #3250). To make the initial fixative, the 32% EM-grade paraformaldehyde was diluted to 4% with the osmo-adjusted dissection buffer.

For processing cochlea, mice were euthanized using CO_2_, and their temporal bones were extracted from the skull and put in a dish with ice-cold dissection buffer, as described in detail by Miller et al.^17^. The inner ears were dissected and transferred to a dish with a fixative, and a hole was poked in the bony cochlear shell at the apex. The fixative was slowly perfused through round and oval windows. The perfused inner ears were then incubated in the fixative for 40 min at RT and further dissected. For the trachea, 3-month-old mice were euthanized using CO2. An incision along the ventral side exposed the torso up to the chin, followed by a second incision along the sternum, allowing the ribs to be opened outward, exposing the trachea. The trachea was cut at the top and the bottom and immediately placed in the dissection buffer. The trachea was cut along its axes to facilitate the antibodies’ access to the internal surface of the trachea, which contains the multiciliate cells. The trachea was treated similar to the auditory epithelium for all subsequent immunofluorescence and SUB-immunogold-SEM steps.

### Primary antibodies

The antibodies used in this study were mouse anti-EPS8 (Clone 15, Fisher Scientific, BDB610143), used at 1:300; rabbit anti-PMCA2a (F2a, gift from P. Barr-Gillespie) used at 1:250^19^; rabbit anti-MYO15A-L (PB888, gift from J. Bird and T. Friedman, used at 1:1000^15^; rabbit anti-ACE2 (Abcam, #ab15348) used at 1:100^30^; rabbit anti-RFP (Rockland, #600-401-379) used at 1:100; mouse anti-acetylated Tubulin (Tuj1) (Sigma, Clone 6–11B-1, #T7451) used at 1:600.

### Immunofluorescence staining and imaging

The whole-mount immunofluorescence staining and imaging of the mouse cochlear HBs in this study followed our previously published detailed protocol^17^. Briefly, the fixed inner ear samples were transferred to new dishes containing PBS, and sequential removal of the bony cochlear shell, stria vascularis, Reissner’s membrane, tectorial membrane, and the modiolus was performed. The finely dissected organs of Corti were transferred to a glass well plate containing PBS and 0.05% Triton X-100 and permeabilized for 20 min at RT. The glass well plate was on an orbital shaker with a 60-rpm speed during permeabilization. After permeabilization, the samples were blocked in PBS with 0.05% Tween 20 (PBST) containing 4% bovine serum albumin Fraction V (BSA) overnight or at least 6 h at 4 °C. The tissues were subsequently incubated with primary antibodies in PBST with 1% BSA (incubation buffer) overnight at 4 °C. After four washes for 5–10 min each, in the incubation buffer at RT, the tissues were incubated with fluorescent dye-conjugated secondary antibodies (Donkey anti-rabbit 488; Thermo Fisher Scientific, #A-10042 or Donkey anti-mouse 647 from Thermo Fisher Scientific, # A-11019) diluted at 1:500 in the incubation buffer at RT for 1–2 h. After one wash with the incubation buffer, the samples were incubated with fluorescent dye-conjugated phalloidin (Invitrogen, #A30104 and #A12379) in the incubation buffer at RT for 25 min. The samples then underwent three washes, 5–10 min per wash, with incubation buffer.

After washing, each sample was mounted on a glass slide under a coverslip using ProLong Gold Antifade Mountant (Thermo Fisher Scientific). Z-stacks were captured using the Airyscan super-resolution mode of a Zeiss LSM880 microscope with Objective C Plan-Apochromat 63x/1.4 Oil DIC M27 lens and Zen black software (Zeiss).

### SUB-immunogold-SEM method

#### Default permeabilization:

After fixation and dissection, the samples were transferred to 2-mL tubes with TBST (150-mM NaCl, 10-mM Tris-HCl, 0.05% Tween-20, pH 7.5). By default, the permeabilization consisted of incubation with 0.05% Triton X-100 in TBST for 20 min at RT under nutation mixing at 5 rpm (Boekel Scientific, variable speed mini orbitron, #201100), followed by a 5-min TBST wash.

#### Dehydration–rehydration permeabilization:

After fixation, the samples were transferred to 2-mL tubes with TBST and placed on ice. The dehydration–rehydration process involved buffer exchange with ice-cold solutions of increasing ethanol percentages in MilliQ water for 5 min without mixing. Liquid transfer was performed with a disposable pasteurette pipette, permanently submerging the sample. The buffer sequence was H_2_O, 15% ethanol, 30%, 50%, 75%, 95%, 100%, 100%, 95%, 75%, 50%, 30%, 15% ethanol, H_2_O, TBST.

#### Blocking:

Samples were blocked in TBST containing 4% BSA for at least 6 h or overnight at 4 °C.

#### Immunogold staining:

The samples were transferred to 0.3-mL PELCO mini vials (TED PELLA, #21441) sealed with parafilm with primary antibodies in TBST with 1% BSA under nutation mixing at 5 rpm, overnight at 4 °C. The samples were transferred into 2-mL tubes with a micro dissecting spoon (Biomedical Research Instruments, #15–1025), always maintained in liquid, rinsed once, and washed thrice for 15 min with 1% BSA TBST. The samples were then transferred to new 0.3-mL PELCO mini vials with 5-nm, 10-nm, or 15-nm gold-conjugated goat anti-rabbit or mouse IgG (BBI: 1:200 in 1% BSA TBST) and incubated overnight at 4 °C. After secondary antibody incubation, the samples were rinsed once and washed thrice for 15 min each with 1% BSA TBST in 2-mL tubes.

#### Post-fixation:

The samples were then rinsed twice with 0.1-M sodium cacodylate buffer (pH 7.2) and fixed with 10% glutaraldehyde and 4% PFA in 0.1-M sodium cacodylate buffer for at least 24 h at 4 °C without agitation.

#### OTOTO post-fixation:

In the case of OsO_4_ (EMS #19150)/OTOTO (EMS #21900) sequential post-fixation, the samples were first transferred to glass vials using a micro dissecting spoon and washed with 0.1-M sodium cacodylate buffer. The samples were fixed with 1% Osmium in 0.1-M sodium cacodylate for 1 h at RT without agitation and protected from light. The samples were then washed four times (5 min each) using 0.1-M sodium cacodylate, then thrice using H_2_O. Subsequently, the samples were incubated with 1% thiocarbohydrazide for 20 min at RT without agitation and protected from light. The samples were then washed four times (5 min each) using water, and then thrice using 0.1-M sodium cacodylate. The sequence was repeated making a total of three osmium and two thiocarbohydrazide incubations.

#### Dehydration:

The samples were washed using 0.1-M sodium cacodylate buffer and transferred to a sample holder for critical drying point in milliQ water. The samples were then dehydrated on ice with ice-cold ethanol solution diluted using milliQ water (15%, 30%, 50%, 75%, 95%, 100%, and 100%, 5-min incubations), with the sample holder permanently submerged. The sample holder was placed immediately in the chamber and processed for critical drying point (Autosamdri-815A, Tousimis). The cochleae were mounted on studs using silver paint and coated with 2- to 3-nm of palladium (sputter coater EMS150TS, Electron Microscopy Sciences) as described by Grillet^18^. The samples were imaged using a 5-kV accelerated voltage and a 100-pA beam current using a concentric BSE detector on an FEI Magellan 400 XHR Field Emission SEM (Stanford Nano Shared Facilities). The microscope is periodically calibrated for measurements using an SIRA-type calibration specimen for ultra-high-resolution modes with a 2% error between 50- and 350-k magnification in our imaging settings. For OTOTO postfixed samples, the beam current was bumped to 200-pA. The gold beads, characterized by their circular shape and defined diameter, were easily identified as strong BSE sources at the cell surface. The micrograph contrast was adjusted or pseudo-colored in postproduction using Photoshop (Adobe) to display the gold better when needed. As stage tilting is impossible in backscattered electron imaging mode, HB orientation could not be adjusted for optimal imaging. The distance measurements of the gold to stereocilia tips were performed using ImageJ2 after scale calibration, placing the measuring ends at the center of the gold bead and the pinnacle of the stereocilia tip. These distances were approximations of the absolute distances, as we measured the shortest distance between the gold beads and the stereocilia tip on 2D pictures without considering the stereocilial volume and the perspective distortion of the images. Conversion of the SEM-determined width and height dimensions were calculated using the shrinkage factor reported by Miller et al.^13^, including the error propagation.

### Quantification and statistical analysis

Measurements were taken from distinct samples. Statistical analyses and sample sizes for all the experiments are detailed in the figure legends and Extended Data Table 1. Normality tests determined whether downstream tests should be parametric or nonparametric. Mann–Whitney *U* tests (one-tailed) were employed for nonparametric pairwise comparisons of two groups. To compare multiple ages, nonparametric Kruskal–Wallis tests followed by Dunn’s multiple comparison tests were performed. Refer to Extended Data Table 1 for a comprehensive group and statistical description. GraphPad Prism 9.4 for Mac (GraphPad Software, San Diego, CA, USA) was used for the statistical analyses.

## Data Availability

Any data presented in the paper is available upon reasonable request by contacting N.G.
